# Modular structure in fish co-occurrence networks: A comparison across spatial scales and grouping methodologies

**DOI:** 10.1371/journal.pone.0208720

**Published:** 2018-12-14

**Authors:** Daniel J. McGarvey, Joseph A. Veech

**Affiliations:** 1 Center for Environmental Studies, Virginia Commonwealth University, Richmond, Virginia, United States of America; 2 Department of Biology, Texas State University, San Marcos, Texas, United States of America; Tuscia University of Viterbo, ITALY

## Abstract

Network modules are used for diverse purposes, ranging from delineation of biogeographical provinces to the study of biotic interactions. We assess spatial scaling effects on modular structure, using a multi-step process to compare fish co-occurrence networks at three nested scales. We first detect modules with simulated annealing and use spatial clustering tests (interspecific distances among species’ range centroids) to determine if modules consist of species with broadly overlapping ranges; strong spatial clustering may reflect environmental filtering, while absence of spatial clustering may reflect positive interspecific relationships (commensalism or mutualism). We then use non-hierarchical, multivariate cluster analysis as an alternative method to identify fish subgroups, we repeat spatial clustering tests for the multivariate clusters, then compare spatial clustering results among modules and clusters. Next, we compare species lists within modules and clusters, and estimate congruence as the proportion of species assigned to the same groups by the two methods. Finally, we use a well-documented nest associate complex (fishes that deposit eggs in the gravel nests of a common host) to assess whether strong within-group associations may, in fact, reflect positive interspecific relationships. At each scale, 2–4 network modules were detected but a consistent relationship between scale and the number of modules was not observed. Significant spatial clustering was detected at all scales for network modules and multivariate clusters but was less prevalent at smaller scales. Congruence between modules and clusters was always < 90% and generally decreased as the number of groups increased. At all scales, the complete nest associate complex was completely preserved within a single network module, but not within a single multivariate cluster. Collectively, our results suggest that network modules are promising tools for studying positive interactions and that smaller scales may be preferable in this research.

## Introduction

Efforts to understand the structural [1,2] and functional [3,4] properties of ecological networks are quickly becoming central themes in community ecology and biogeography. This trend has been driven by a fundamental need to understand how perturbations may propagate through interconnected ecosystems, as well as the enhanced availability of large datasets to represent complex networks [5–9]. In a network graph, distinct entities–usually individuals or species–are represented as nodes or vertices and connections among entities are represented as links or edges. These connections often represent species’ co-occurrences [10,11] or food web links between predators and their prey [12,13] when working with unipartite or ‘one-mode’ networks. Connections within bipartite or ‘two-mode’ networks may also represent associations between species with distinct functional roles, such as plants and their pollinators [14,15], or species’ presences within a matrix of potential habitats [16,17].

One aspect of network structure that is particularly relevant to many ecological questions is modularity. Modularity is the tendency for networks to consist of highly interconnected sub-groups of species’ nodes that are distinguished from other such groups, or modules, by relatively sparse among-group connections [18]. Depending on one’s interest, network modules can serve several purposes. At regional to continental scales, modularity analysis of bipartite networks (species × site data) can be used to detect biogeographical provinces and may be a superior alternative to multivariate ordination and clustering algorithms [19]. Multivariate methods that use species’ presence-absence matrices as raw data can be sensitive to the choice of a particular dissimilarity index and to the rules used to combine or agglomerate entities within clusters [20]. But bipartite modularity values are derived solely from observed connections between species and sites, and do not incorporate an abstract measure of dissimilarity [17,18].

At smaller scales (e.g., forest plots and small lakes), modules are used to study the structure and stability of interactive communities. Previous work built upon well-documented, empirical examples of antagonistic and mutualistic interactions within unipartite [21,22] and bipartite networks [14,23]. However, network tools are now being used to infer biotic interactions when direct, observational information on species’ interactions is incomplete [11,24,25]. For instance, when working with unipartite co-occurrence networks, it is reasonable to predict that positive interspecific interactions are more likely to exist within modules than among modules [16]. In this way, modularity analysis may be a logical precursor to experimental tests of species’ interactions [10].

With network modules now being used for such diverse purposes as detecting biogeographical regions and characterizing species’ interactions, it is increasingly necessary to account for the effect of spatial scale [7,24]. For example, strong modular structure within a unipartite co-occurrence network may reflect positive interactions among species within shared modules and/or negative interactions among species in different modules. However, if modularity analyses use species co-occurrence data that are aggregated across large spatial extents that exceed individual species’ ranges, frequent co-occurrence within a shared module may be mistaken for a positive interaction when it is in fact the result of environmental filtering [11]. Filtering of species among different habitat types will tend to place species with similar habitat requirements in close proximity, resulting in frequent co-occurrences that may be mistaken for positive interactions [26,27]. Alternatively, if environmental filtering or historical barriers to species’ movements create disjunctions in species’ ranges, strong modular structure may be misinterpreted as evidence of negative interspecific interactions among species in different modules [28].

In this study, our primary objective is to quantify the effect of spatial scale on perceived modular structure within unipartite networks. Secondary objectives are to determine whether network modules partition species into subgroups in a fundamentally different way than multivariate clusters, and to test if differences between modules and clusters are themselves scale dependent. The later objectives are motivated by recent efforts to assess whether network-based algorithms for detecting subgroups within ecological communities are more effective than multivariate clustering algorithms [17,29,30].

To accomplish these objectives, we build co-occurrence networks for Mississippi River (USA) fish assemblages at three nested spatial scales. At each scale, we then use a three-step process to examine network modules and to assess scale dependence. First, we delineate network modules and compared the modules with clusters identified by a non-hierarchical clustering algorithm. These module vs. cluster comparisons test for congruence in the numbers of fish species that are assigned to the same groups when modularity and cluster analysis are used. Second, we test for ‘spatial clustering’ within network modules and multivariate clusters. Our intent is to determine whether modules and/or clusters are comprised of species with broadly overlapping ranges; if so, they may constitute distinct regional species pools or biogeographical units (*sensu* environmental filtering), rather than subsets of species joined by interactions *per se*. Third, we use a nest associate complex–fishes that deposit their eggs within the pebble mound nests of a common host species–as an empirical benchmark to determine whether network modules and/or multivariate clusters consistently assign members of the complex to the same group. This third step is of particular interest because past efforts to infer biotic interactions from co-occurrence data have often been constrained by a lack of corroborating, empirical evidence of real-time interactions [11,31] and by ambiguity in the specific types of interactions that one seeks to identify [24,32,33]. Interactions may be positive or negative, but they may also be symmetric or asymmetric. For instance, mutualism and commensalism are both positive relationships, but the former is symmetric, the later asymmetric. By using the nest associate complex, we focus on a specific interaction that is well-documented and precisely defined as positive symmetric, or mutualistic [34–36]. Finally, we compare results from each of the three steps to determine whether they vary in a consistent manner among spatial scales.

## Materials and methods

### Fish data within nested spatial units

Fish occurrence data were obtained from a large database of Mississippi River Basin fish assemblage samples, collected by the U.S. Environmental Protection Agency. We combined Mississippi Basin samples from the Environmental Monitoring and Assessment Program [37] and the National Rivers and Streams Assessment [38] databases. These two programs utilized similar, standardized field methods (single-pass backpack electrofishing surveys) that were calibrated with sampling effort curves (i.e., plotting the number of sample units needed to detect 95% of all locally occurring species) to ensure samples would be comparable among different sites [39–41]. Together, these databases provided occurrence records for 300 fish species distributed among 1018 Mississippi River Basin sites. Samples were organized at three spatial scales: the entire Mississippi Basin and two smaller, nested scales. We used 2-digit hydrologic units (HU-2) from the Watershed Boundary Dataset [42] to represent ‘medium’ sized river basins (mean size within Mississippi Basin = 231,968 km^2^) and 4-digit hydrologic units (HU-4) to represent ‘small’ basins (mean size within Mississippi Basin = 26,190 km^2^). The complete Mississippi River Basin was treated as a 0-digit hydrologic unit (HU-0; size = 3,247,552 km^2^).

### Co-occurrence networks, network modules, and clusters

**Fig 1:**
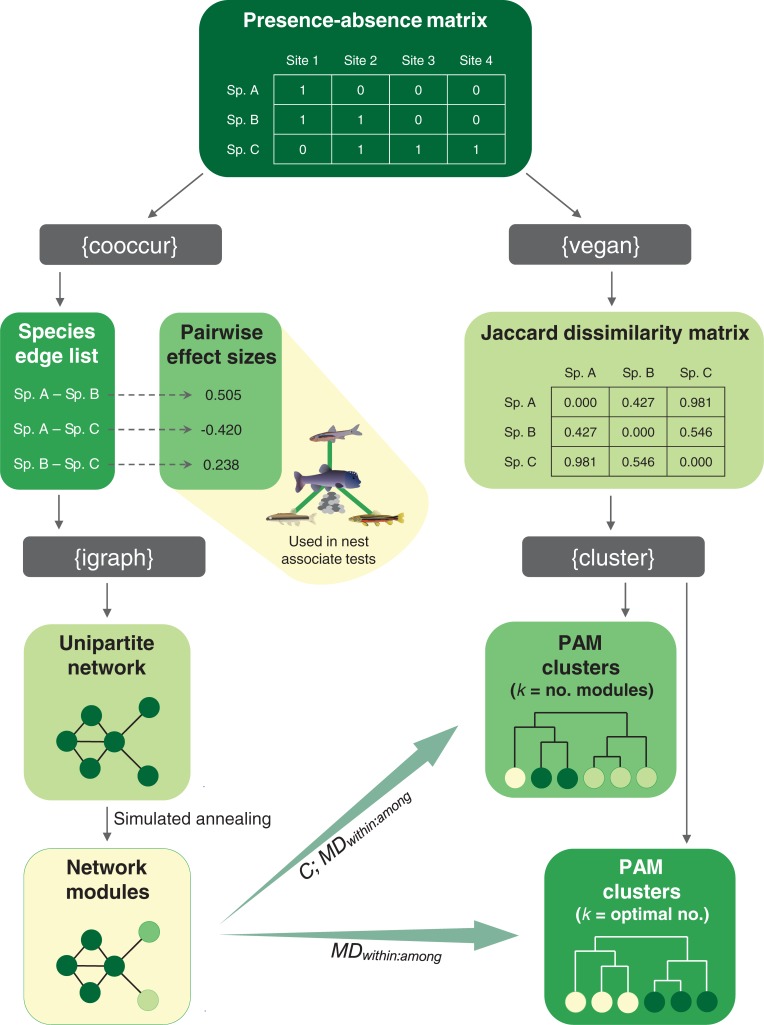
Workflow diagram of the network and cluster analyses. For each of the 10 nested river basins included in the study, a species’ presence-absence matrix was first compiled then converted to a species’ edge list or a species × species Jaccard dissimilarity matrix. Edge lists were used to build unipartite networks, followed by modularity analysis through simulated annealing. (Pairwise effect sizes from cooccurrence analyses were estimated for use in subsequent tests of nest associate species and are not utilized within the workflow diagram.) Dissimilarity matrices were used in PAM cluster analyses. Comparisons of network modules and PAM clusters included congruence (*C*) analysis and ratios of mean distances within and among groups (*MD*_*within*:*among*_) when the number of PAM clusters (*k*) was equal to the number of modules (*k* = no. modules). However, network and cluster comparisons were limited to *MD*_*within*:*among*_ when an optimal number of PAM clusters was independently identified with the gap statistic (*k* = optimal no.). R packages used in each step of the workflow are shown in curly brackets within gray bubbles (e.g., ‘igraph’).

Fish co-occurrence networks were built for the complete Mississippi Basin and for each of the nested sub-basins that included a sufficient number of sampling sites (*n* ≥ 70 for HU-2 basins; *n* ≥ 35 for HU-4 basins). Site × species occurrence matrices (species’ presence-absence matrices) were first converted to species × species edge lists (two-column lists of species pairs that co-occur at one or more sites) using the *cooccur* package in R [43]. Edge lists were then used to build unweighted, unipartite networks in R package *igraph* [44]. A workflow diagram of all data conversions and analyses is shown in Fig 1.

Network modules were identified with a simulated annealing algorithm [45]. Specifically, we used the ‘cluster_spinglass’ function (100 spins, start temperature = 1, stop temperature = 0.01, cooling factor = 0.99) in *igraph*. This function calculated an optimal modularity value for each network and partitioned species among distinct groups or modules, then exported the number of detected modules and lists of species membership within each module for further analysis. Simulated annealing was chosen because it generally outperforms other graph partitioning methods [46,47] and the computational burden in working with the modest sized fish network datasets was acceptable (< 10 minutes processing time for each network).

Next, multivariate cluster analysis was used as a second, alternative method to partition species among groups. We first calculated a Jaccard dissimilarity matrix for each of the original site × species occurrence matrices using the ‘vegdist’ function in R package *vegan* [48]. The dissimilarity matrices and the robust ‘partitioning around medoids’ (PAM) alternative to traditional *k*-means clustering [49], as implemented with the ‘pam’ function in R package *cluster* [50], were then used to partition species among clusters (see Fig 1). Notably, two strategies were used to specify the number of PAM clusters, *k*, for each of the fish datasets. First, *k* was made equal to the number of modules detected within a given network, so that direct tests of congruence in species’ groupings among *k* modules and an equivalent number of PAM clusters could be performed (see next paragraph). Second, we used the gap statistic [51] to infer an optimal number of PAM clusters for each fish dataset and made *k* equal to the optimum. This latter method avoided potential circularity in the specification of *k* in cluster analyses, which could generate bias in spatial clustering tests (see ‘spatial clustering’ below) if the optimal number of PAM clusters within a given fish dataset was smaller or larger than the number of modules within the same dataset. However, the second method did not allow us to perform direct tests of congruence between module and cluster assignments (see next paragraph) because the numbers of modules and clusters tended to differ.

**Fig 2:**
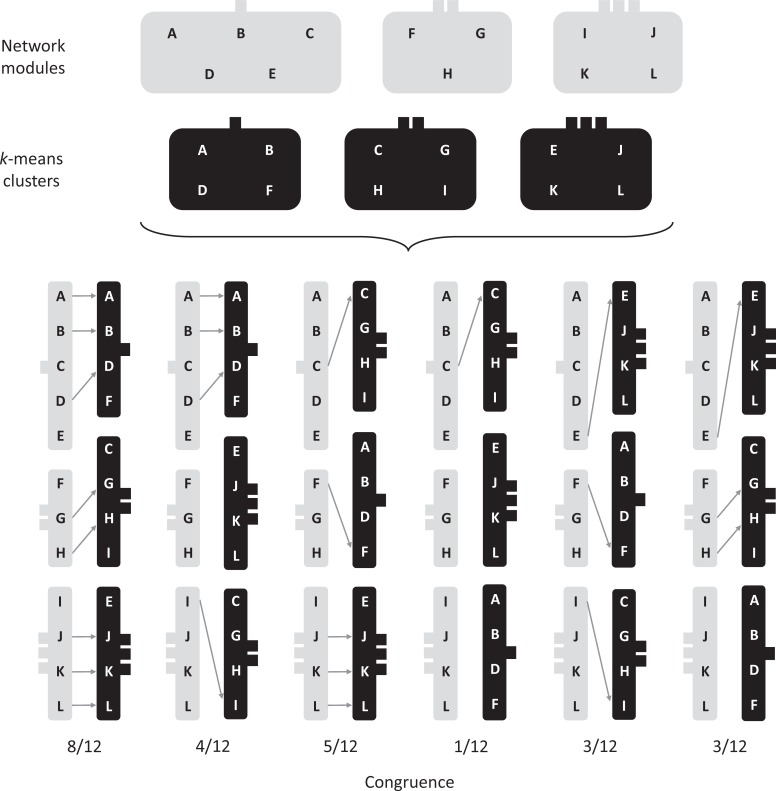
Illustration of the process used to quantify congruence (*C*) among network modules and PAM clusters. Hypothetical results are shown at the top of the diagram for network modules and PAM clusters: the same 12 species (A—L) were first partitioned among three network modules (grey boxes), then among three PAM clusters (black boxes). Partitioning of species among network modules and PAM clusters was conducted independently, though the number of PAM clusters was determined by (i.e., equivalent to) the number of network modules identified by the simulated annealing algorithm. Note that the number of species assigned to each module and cluster may vary and is determined by the annealing and clustering algorithms respectively. In this hypothetical example, species numbers are variable among modules, with five, three, and four species assigned to the first, second, and third modules respectively, but four species are assigned to each of the three clusters. Congruence between network modules and PAM clusters is based on the number of instances in which species are grouped together in the same module and cluster. For example, in the first pair of columns shown at the lower-left side of the diagram, a total of eight species are assigned to the same module and cluster groups, as indicated by gray arrows. Congruence in this instance is calculated as eight matches divided by the total number of species (i.e., *C* = 8 ÷ 12 = 0.67). To aid in interpretation, modules and clusters are identified by the number of ‘tabs’ assigned to each; one, two, or three tabs per module or cluster are shown and are consistent among the upper and lower parts of the diagram (e.g., the black cluster with two tabs consistently contains species C, G, H, and I). When assessing *C*, it is critical to recognize that the labels used to identify network modules and PAM clusters (one, two, or three tabs in this illustration) are arbitrary. Only the shared identities of the species’ lists within each module or cluster are important. Therefore, a complete test of *C* cannot be performed by simply comparing module 1 vs. cluster 1, module 2 vs. cluster 2, etc. Rather, the level of congruence between modules and clusters must account for multiple module vs. cluster combinations. This is achieved by ‘rotating’ the clusters in a combinatorial manner, as shown in the 2^nd^ through 6^th^ pairs of columns in the lower part of the diagram. The goal is to investigate all possible combinations of modules and clusters while searching for the *highest possible* level of *C*, given the constraint of the observed species’ assignments within modules and clusters (shown at top of diagram). Note, however, that the network modules do not need to be rotated during the combinatorial comparisons with the PAM clusters, as the objective is to assess the degree to which species’ assignments within clusters match species’ assignments within modules. Thus, with a system of three modules and three clusters, six cluster rotations are needed to explore all possible module vs. cluster combinations. The observed *C* value for each of the six rotations is shown at the bottom of the diagram. Hence, the first combination of modules and clusters (i.e., first two columns at lower-left) in this illustration leads to the highest possible *C* value which is then taken as the overall amount of congruence between the modules and clusters.

Congruence (*C*) between species’ assignments within network modules and PAM clusters was quantified for each dataset with the procedure shown in Fig 2, while *C* significance levels were estimated with a permutation test. In each of 999 permutations, we randomized species’ assignments within PAM clusters and re-calculated *C* between the observed network modules and the permuted clusters. A *p*-value for *C* was then estimated as the proportion of permutations in which the randomized *C* value was equal to or greater than the observed *C* value. All *C* tests were performed with a custom Visual Basic for Applications function in Microsoft Excel.

### Spatial clustering

Spatial clustering tests focused on the spatial orientations of nodes within and among network modules when node spatial positions were estimated as the centroids of the respective species’ ranges (see next paragraph and Fig 1A). We reasoned that if species within the same modules are positioned closely together in space, relative to species in other modules (Fig 1B), then explanations for modular structure that invoke positive species interactions within modules, such as commensalism or mutualism, would be weakened. In this spatial clustering scenario, modular structure could be parsimoniously explained by historical events or environmental filtering processes that lead to overlapping species ranges. Alternatively, if no spatial clustering of species within modules is observed (Fig 1C), then shared module membership may indeed reflect positive or facilitative interactions among species. We also assumed that spatial clustering will be most prevalent at the largest spatial scales, where spatial clusters will tend to represent distinct biogeographic provinces, and least prevalent at the smallest scales. We therefore performed spatial clustering tests at each of the three nested scales (HU-0, 2, and 4) and tested for spatial clustering among PAM clusters as well as modules.

**Fig 3:**
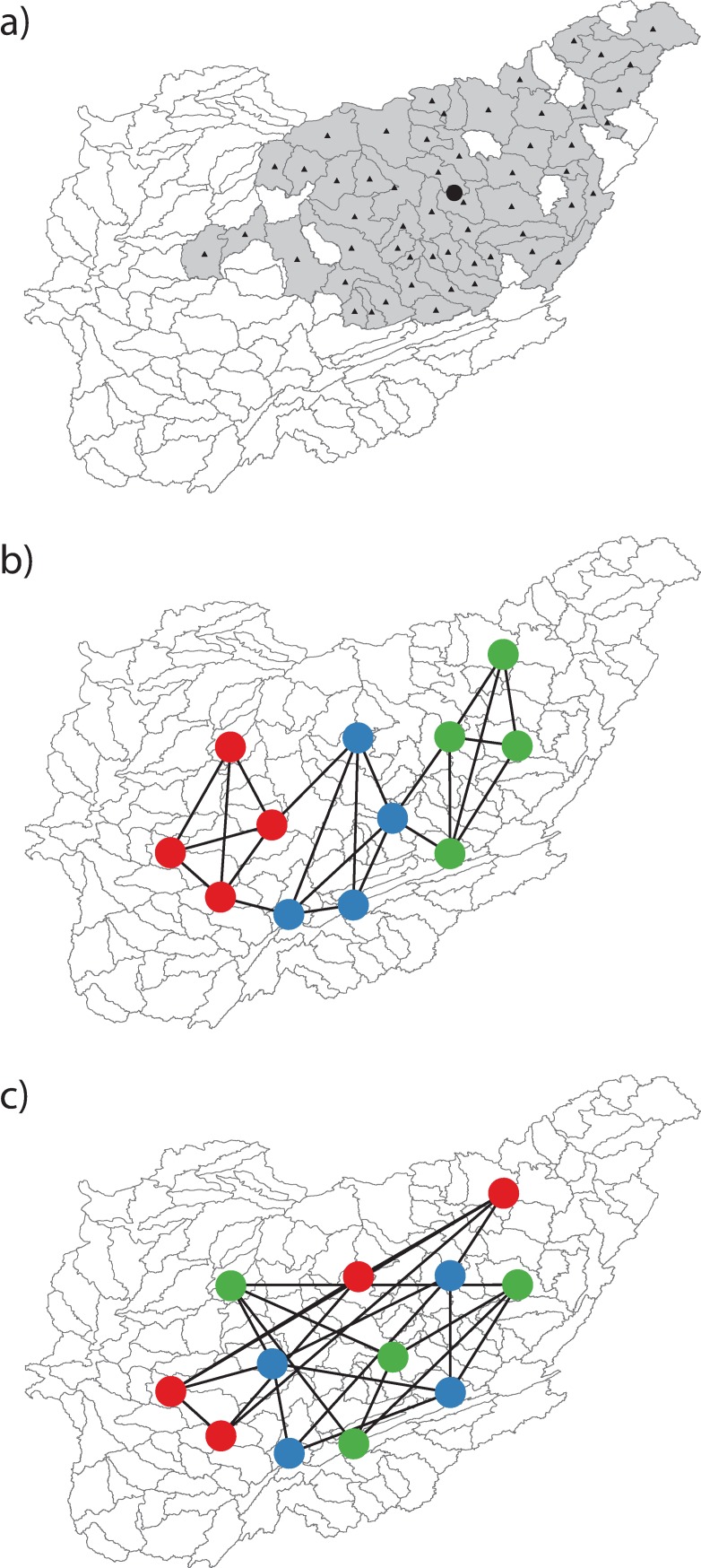
Hypothetical maps of fish co-occurrence networks. These maps demonstrate spatial clustering and the absence of spatial clustering. Each map is centered on the Ohio River Basin with 8-digit hydrologic units delineated by grey lines. Panel **a** illustrates the process used to interpolate range centroids for individual species. In this example, the native range of the Variegate Darter (Etheostoma variatum) is indicated by shaded grey polygons (see main text for source data). The center or ‘centroid’ of each range polygon, interpolated in a two-dimensional Cartesian plane, is indicated by a black triangle. The master centroid of the species’ native range, calculated as the grand mean of the *x* and *y* coordinates for individual range polygon centroids, is shown as a black circle. Panel **b** illustrates a hypothetical network of 12 fish species, partitioned into three distinct network modules (red, blue, and green circles). In this instance, strong spatial clustering is evident. Panel **c** illustrates a similar fish network, but one that is characterized by a lack of spatial clustering.

To perform spatial clustering tests, we first located the range centroid for each species that was included in one of the co-occurrence networks. We started by importing and analyzing species’ distribution data from NatureServe [52] in a geographic information system. The NatureServe fish database combines fish occurrence records (point samples) from published research and state-sponsored natural heritage programs, then uses these records to document species’ occurrences within small drainages (mean area within Mississippi River Basin = 4022 km^2^), as represented by 8-digit hydrologic units (HU-8; USGS 2013). We used the ‘Feature to Point’ tool in ArcGIS 10.1 (Environmental Systems Research Institute, Redlands, CA) to interpolate the spatial centroid of every HU-8 within the Mississippi Basin. We then queried all HU-8’s from the NatureServe [52] database with known occurrence of a given species and calculated the overall range centroid as the mean *x* coordinate and the mean *y* coordinate among all HU-8 occurrence centroids. This interpolation process is illustrated in Fig 3A and was repeated at each of the three nested scales for every species included in one of the co-occurrence networks. Prior to interpolation, all spatial data were converted to Albers equal-area (NAD83) conic projection.

Next, we used the species’ range centroids to calculate mean distances (*MD*) among species within groups (*MD*_*within*_) and mean distances among groups (*MD*_*among*_) for all pairwise combinations of species. For each of the nested fish datasets, *MD*_*within*_ and *MD*_*among*_ were independently calculated for network modules and for PAM clusters, both when cluster *k* was equal to the number of network modules and when an optimal *k* was determined with the gap statistic. Our process was modeled after the mean similarity study of [53]. However, we calculated *MD*_*within*_ and *MD*_*among*_ as true Euclidian distances in units of km (see Fig 3), rather than unitless dissimilarity index (e.g., Jaccard dissimilarity) values. Significance levels were then estimated with the multi-response permutation procedure of Mielke et al. [54]. In each of 999 permutations, we randomly shuffled species among groups (network modules or PAM clusters) then recalculated *MD*_*within*_ and *MD*_*among*_ for the permuted data. These permutations focused solely on the average spatial proximities of species within and among groups, without invoking a more liberal process of simulating species’ ranges (i.e., randomizing species’ occurrences within HU-8 units; see Fig 3A) then re-interpolating the range centroids. Finally, a spatial clustering *p*-value was calculated as the proportion of permutations in which *MD*_*within*_ ≥ *MD*_*among*_. This tested the null hypothesis that within-group distances were, on average, smaller than among-group distances (i.e., *MD*_*within*:*among*_ ratios < 1), indicative of spatial clustering. Spatial clustering tests were performed with the ‘meandist’ and ‘mrpp’ functions in *vegan*.

### Nest associates

Nest association within freshwater fish assemblages is a specialized (but not uncommon) reproductive strategy in which associate species seek out and deposit their eggs in pebble mound nests built by a host species [55]. At a minimum, this relationship constitutes asymmetric commensalism; associate species incubate their eggs in well-sorted substrates with superior aeration and parental guarding, at no cost to the nest host [56,57]. But in many instances, this relationship may constitute symmetric mutualism, as egg survival is maximized for nest-building species through a predatory dilution effect [36]. One specific, well-documented example of nest association in streams of the eastern Mississippi River Basin see [34,35,58]) is the connection between the relatively large-bodied, nest-building Bluehead Chub (*Nocomis leptocephalus*) and six species of smaller-bodied minnows (family Cyprinidae), including Rosyside Dace (*Clinostomus funduloides*), Mountain Redbelly Dace (*Chrosomus oreas*), Central Stoneroller (*Campostoma anomalum*), White Shiner (*Luxilus albeolus*), Rosefin Shiner (*Lythrurus ardens*), and Crescent Shiner (*Luxilus cerasinus*).

Focusing exclusively on the river basin in which Bluehead Chub nest associates have been most carefully studied–the Kanawha River Basin (HU-4 scale), nested within the Ohio River Basin (HU-2 scale) and Mississippi River Basin (HU-0 scale)–we used the nest associate complex to evaluate the network analysis results in two ways. First, we assessed the degree to which the nest associate complex was preserved within network modules and PAM clusters (*k* = number of modules and the optimal number of clusters), at each of the three nested spatial scales. The underlying logic was that the majority or entirety of the nest associate complex should be preserved within a single group if modules or clusters are comprised of species linked by positive associations.

Second, we calculated an effect size for every pairwise link within a given network, then compared effect sizes that were exclusive to the nest associate complex with average effect sizes from the complete networks. This tested the hypothesis that links between nest associates would be among the strongest in the network. Pairwise effect sizes were calculated directly from the raw fish occurrence data, using the probabilistic co-occurrence model of Veech [59,60]. Briefly, this model uses a combinatorics approach to calculate the probability that two species will co-occur at *j* sites, then to compare this expected value with an observed co-occurrence value. Observed co-occurrence values that exceed the expected value by a significant margin provide evidence of positive associations and vice-versa. Following Veech [60], effect sizes were calculated as the differences between observed and expected co-occurrences for each pairwise association, using R package *cooccur*.

### Data and code

Complete fish occurrence data, species’ range centroids, R code to reproduce all network and cluster analyses, and the Visual Basic for Applications function used in congruence tests are available on Figshare at https://doi.org/10.6084/m9.figshare.c.4151780.v1

## Results

Sample sizes within basins were adequate to build four fish co-occurrence networks at the HU-2 scale and five at the HU-4 scale, in addition to the HU-0 scale network for the entire Mississippi River Basin (Table 1). The number of modules detected in each network ranged from 2–4 and was not a clear function of spatial scale, as the minimum and maximum number of modules were both associated with the smallest (HU-4 scale) networks.

**Table 1:** Congruence (*C*) and spatial clustering results for fish network modules and partitioning around medoids (PAM) clusters.

				Spatial clustering						
				Network modules		PAM clusters				
						*k* = no. modules		*k* = optimal no.		
Scale	River basin	Modules	*C*	*MD*_*within*:*among*_	*p*	*MD*_*within*:*among*_	*p*	GS no.	*MD*_*within*:*among*_	*p*
HU-0	Mississippi	4	0.50	0.75	0.001	0.75	0.001	3	0.83	0.001
HU-2	Ohio	4	0.51	0.85	0.001	0.76	0.001	4	^a^	
HU-2	Upper Mississippi	4	0.69	0.80	0.001	0.82	0.001	3	0.90	0.005
HU-2	Missouri	3	0.65	0.54	0.001	0.67	0.001	2	0.60	0.001
HU-2	Arkansas-White-Red	3	0.73	0.79	0.001	0.74	0.001	2	0.76	0.001
HU-4	Allegheny	3	0.80	1.02	0.372	1.07	0.185	1	^b^	
HU-4	Upper Ohio	2	0.88	0.85	0.001	0.86	0.001	4	0.79	0.001
HU-4	Kanawha	4	0.48	0.88	0.001	0.75	0.001	2	0.82	0.001
HU-4	Upper Tennessee	4	0.56	0.97	0.030	0.97	0.078	2	0.98	0.139
HU-4	Missouri	3	0.61	0.89	0.043	1.07	0.933	3	^a^	

Results are shown at three spatial scales, corresponding to hydrologic units (HU-0, -2, -4). *Modules* is the number of distinct network modules detected with simulated annealing for each fish network. *C* is the maximum proportion of fish species that were assigned to the same groups by modularity and PAM cluster analyses, when the number of PAM clusters (*k*) was equivalent to the number of network modules. *MD*_*within*:*among*_ is the ratio of the mean Euclidian distance (between species' centroids; see main text) within and among groups. *MD*_*within*:*among*_ values are shown with permutation test (999 iterations) *p*-values for network modules, for PAM clusters when *k* was equivalent to the number of modules, and for PAM clusters when *k* was equal to the optimal number of clusters identified with the gap statistic. *GS no*. is the number of PAM clusters detected when the gap statistic was used.

^a^ Values not reported because the optimal number of PAM clusters was equal to the number of network modules and spatial clustering test results were therefore identical.

^b^ Values not reported because the optimal number of PAM clusters was one (i.e., among group comparisons were not possible).

Congruence in species’ membership among network modules and an equivalent number of PAM clusters (*k* = no. modules) was variable, ranging from 0.48–0.88, and generally decreased as the number of modules increased (Table 1). This inverse trend between *C* and the number of modules was intuitive because placement of species within the same groups is, on average, less likely when the number of groups is larger. For all networks, the *C* permutation test results were highly significant. In no instance did the randomized *C* value exceed the observed *C* value (*p* < 0.001 in all permutation tests; not shown in Table 1). Thus, we found no evidence to support the hypothesis that the observed *C* values can be attributed to random partitioning of species among an equivalent number of network modules and PAM clusters.

Spatial clustering tests revealed a high level of clustering at each of the three spatial scales. For network modules, *MD*_*within*_ was significantly (*p* < 0.05) smaller than *MD*_*among*_ (i.e., *MD*_*within*:*among*_ ratios < 1) in 9 of 10 basins (Table 1). For PAM clusters, *MD*_*within*:*among*_ ratios were significantly < 1 in 7 of 10 basins when *k* was equal to the number modules (*k* = no. modules; Table 1), and in 6 of 7 basins when an optimal *k* value was determined with the gap statistic (*k* = optimal no.; Table 1). Notably, the prevalence and magnitude of spatial clustering did vary among scales. At the smallest HU-4 scale, highly significant spatial clustering (*p* ≤ 0.005) was detected in only 2 of 5 river basins (Upper Tennessee and Missouri). At the larger HU-0 and HU-2 scales, highly significant (*p* ≤ 0.005) spatial clustering was detected in all river basins and *MD*_*within*_:*MD*_*among*_ ratios were consistently smaller than at the HU-4 scale. In all cases, spatial clustering test results were generally similar among network modules and PAM clusters; *MD*_*within*_:*MD*_*among*_ ratios and *p*-values did not deviate strongly among modules and clusters (inclusive of both *k* = no. modules and *k* = optimal no. results) when comparing results for a given river basin (Table 1).

**Fig 4:**
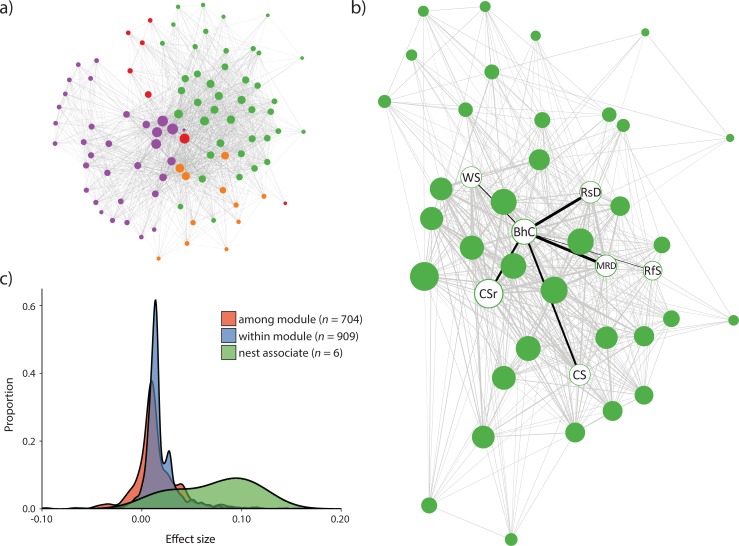
The Kanawha River Basin (HU-4 scale) fish network. Panel **a** shows the complete network of 94 fish species, with co-occurrences among species indicated by light grey edges and the four distinct modules indicated by node colors. The network was plotted with the Kamada-Kawai force-directed layout, which positions the most highly connected nodes near the center and weakly connected nodes along the periphery. (Note that this network does not incorporate species’ centroids in the layout.) Panel **b** magnifies the Kanawha River network module (green nodes) that includes the Bluehead Chub (‘BhC’; *Nocomis leptocephalus*) and its six known nest associates: White Shiner (‘WS’; *Luxilus albeolus*), Rosyside Dace (‘RsD’; *Clinostomus funduloides*), Mountain Redbelly Dace (‘MRD’; *Chrosomus oreas*), Rosefin Shiner (‘RfS’; *Lythrurus ardens*), Crescent Shiner (‘CS’; *Luxilus cerasinus*), and Central Stoneroller (‘CSr’; *Campostoma anomalum*). Edge widths within the green module are proportional to the effect sizes estimated with the probabilistic model of co-occurrence (see main text) and nest associate pairs are highlighted by black edges. Panel **c** illustrates density functions (kernel estimates) for three groups of co-occurrence effect sizes within the Kanawha River fish network: all edges between species in different modules (‘among module’), all edges between species in the same modules (‘within module’), and a group that is exclusive to the six edges between Bluehead Chub and each of its nest associates.

Analysis of the nest associate data suggested that network modules may outperform PAM clusters when the objective is to detect groups of species that are linked by positive interactions. At each of the three spatial scales, the complete nest associate complex (i.e., the Bluehead Chub and its six associates) was preserved in a single network module (Table 2). However, when species were partitioned among PAM clusters, results were more variable. At the HU-0 scale, all members of the nest associate complex were assigned to the same cluster. But at the smaller HU-2 and HU-4 scales, only 2 of 6 and 3 of 6 nest associates were included in the same cluster as the Bluehead Chub, respectively, when the number of PAM clusters was equal to the number of network modules. When an optimal number of PAM clusters was used (two clusters rather than four; see Table 1), all of the nest associate species were again included in the same cluster. In Fig 4, we illustrate the complete HU-4 scale fish network for the Kanawha River (panel a), with distinct modules indicated by node colors. Links between the Bluehead Chub and its nest associates are highlighted in Fig 4B, where we isolate and magnify the module that contains the nest associate complex.

**Table 2:** Representation of the Bluehead Chub (*Nocomis leptocephalus*) nest associate complex at three spatial scales.

		Completeness				
			PAM cluster		Effect size	
Scale	River basin	Network module	*k* = no. modules	*k* = optimal no.	Nest associates only (1 s.d.)	All species in network (1 s.d.)
HU-0	Mississippi	1.00	1.00	1.00	0.007 (0.004)	0.001 (0.005)
HU-2	Ohio	1.00	0.33	0.33	0.027 (0.015)	0.002 (0.010)
HU-4	Kanawha	1.00	0.50	1.00	0.072 (0.036)	0.016 (0.021)

At each of the three scales (HU-0, -2, -4), the completeness of the nest associate network (i.e., the proportion of the six known Bluehead Chub nest associates that was included in the same module or cluster as the Bluehead Chub) is shown for network modules and for PAM clusters. Separate completeness results are shown for PAM clusters when the number of clusters was equal to the number of network modules and when an optimal number of clusters was determined with the Gap Statistic (see main text). Mean effect sizes and standard deviations (s.d.) from the probabilistic co-occurrence model are also shown at each scale for species-pairs that were limited to the Bluehead Chub nest associates and for all species-pairs within a given network.

Comparisons of co-occurrence effect sizes indicated that the ‘signal’ of the nest association is strongest at the smallest scale. The mean effect size between the Bluehead Chub and each of its six nest associates decreased by a large margin with successive increases in scale (Table 2). But at each scale, the mean effect size of the nest associate complex was much larger than the mean effect size when calculated for all species pairs within the network. For instance, in the Ohio River Basin (HU-2 scale), the mean effect size within the nest associate complex (0.027) was approximately one order of magnitude greater than the mean effect size among all species pairs within the network (0.002; see Table 2).

## Discussion

### Spatial scale and network modularity

Network analyses are now common in ecological and biogeographical research, yet many basic questions on methodology and scale dependence remain unanswered [24,28]. We examined the effect of spatial scale on network modularity, using freshwater fishes within the Mississippi River Basin as a case study. Specifically, we tested for spatial clustering within network modules at three spatial scales to determine whether modular patterns reflect a combination of overlapping species’ ranges within modules and disjunct species’ ranges among modules. In this way, we used spatial clustering as a null model for tests of biotic interactions within modules. It is perhaps logical to expect that the dense associations *within* network modules reflect positive interactions such as commensalism or mutualism, while the sparse associations *among* modules reflect negative interactions, such as amensalism or competition. But if spatial clustering within modules is strong, as indicated by *MD*_*within*:*among*_ ratios < 1, then explanations for modular structure that invoke biotic interactions may be unjustified. In such scenarios, positive co-occurrence may simply be due to environmental filtering (e.g., shared habitat preferences) rather than direct positive interactions.

Significant spatial clustering (*p* < 0.01) was detected at all three spatial scales (Table 1). However, the strength of the clustering was variable and declined at smaller scales, as indicated by larger *MD*_*within*:*among*_ ratios for HU-4 scale networks. Significant clustering was also least common for HU-4 scale networks; only 2 of 5 HU-4 scale networks exhibited significant clustering at *p* < 0.01. These observations matched our initial prediction that spatial clustering would be least pronounced at the smallest scale. By extension, they were consistent with the hypothesis that inferences regarding species’ interactions are less likely to be confounded with non-interactive processes, such as environmental filtering, when network analyses are conducted at smaller spatial scales.

Interestingly, the number of modules detected within each river basin was not a clear function of spatial scale. At each scale, a comparable number of modules, usually 3–4, were detected (Table 1). This was surprising because the river basins were spatially nested; HU-4 scale rivers were nested within the HU-2 rivers and each of the HU-2 rivers was nested within the HU-0 scale Mississippi River Basin. If modules within the HU-4 basins are truly discrete subcommunities that share few links with each other, they might well be preserved at larger spatial scales, leading to an additive increase in the number of modules at larger scales. For instance, three, two, and four modules were detected in the HU-4 scale Allegheny, Upper Ohio, and Kanawha River Basins, respectively, and each was nested within the HU-2 scale Ohio River Basin. Thus, a reasonable expectation would have been that the Ohio River fish network would include more than four modules. The fact that it did not warrants a cautious approach when using modules to infer species’ interactions, but it may also be an artifact of species’ occurrences in more than one of the HU-4 scale rivers. For example, 81 fish species occurred in both the Allegheny River and the Kanawha River. Accordingly, each of these species was featured more than once in the HU-4 scale analyses (once per network × multiple networks), but only once in the HU-2 scale Ohio River network. Because each species within a network must be assigned to a single module, it is difficult to anticipate how transitions between nested scales will affect the number or composition of network modules. We therefore suggest that a hierarchical algorithm capable of detecting submodules within modules, such as map equation [61,62], would be a useful next step in the analysis of the fish co-occurrence networks.

### Network modules vs. multivariate clusters

Several authors have compared network modules with multivariate clusters, assessing whether one method provides unique insight or otherwise outperforms the other. For instance, in a continental scale study of Australian plants, Bloomfield et al. [30] concluded that network modules were more effective than multivariate clusters in detecting biogeographical regions, as well as fine-scale transition zones among regions. Similarly, Vilhena and Antonelli [29] reported that network modules were superior to multivariate clusters when searching for biogeographical regions at continental to global scales. Carstensen et al. [17] refrained from labeling one method as superior to the other but did offer a fundamental distinction: ‘Whereas the distance-based clustering methods group [species] according to calculated distances between pairs of [species], the network approach seeks to account for the entire link structure of the network by minimizing links between modules.’

We built upon previous comparisons of network modules and multivariate clusters in two novel ways. First, we repeated the spatial clustering tests from the network modules for the PAM clusters. *MD*_*within*:*among*_ ratios were compared between modules and clusters (in a given river basin) to determine whether one method is more prone to detect groups of species with spatially aggregated ranges than the other. When the number of PAM clusters *k* was constrained to match the number of modules detected within a given fish co-occurrence dataset, we observed no major differences in spatial clustering results. *MD*_*within*:*among*_ ratios and spatial clustering *p*-values were similar for modules and clusters when compared among the 10 nested river basins (Table 1). Furthermore, these similarities were preserved when the gap statistic was used to identify an optimal, unconstrained number of PAM clusters. These results suggest that network modules are neither more nor less likely to identify groups of spatially clustered species than multivariate clusters.

Second, we measured congruence *C* in the numbers of species that were assigned to the same groups by modularity and clustering algorithms. To our knowledge, this is the first study to focus explicitly on species’ lists within modules and clusters, and to compare lists between methods (see Fig 2). We found that species are not partitioned among network modules and PAM clusters in the same way. Across all spatial scales and river basins, median *C* in individual species’ assignments among modules and clusters was 0.64 and *C* never exceeded 0.80 when at least three groups were being compared (Table 1). Results from the *C* tests should be interpreted with caution, as they were only performed for PAM clusters when *k* was equal to the number of modules. We did not calculate *C* when the numbers of modules and clusters (based on the gap statistic optimal *k*) differed because in these situations *C* = 1 was, by definition, an impossible outcome. In this way, we have addressed the question ‘how closely does species composition of PAM modules match species composition in network modules’ without evaluating the opposite question (i.e,. calculating *C* when the number of modules is constrained to equal *k* from an optimal PAM clustering solution). Nevertheless, we have shown that when species are partitioned among network modules, species’ lists within modules will tend to differ, sometimes by a large margin, from group lists that would be obtained with a multivariate clustering algorithm.

Our *C* test results are particularly relevant in a regional species pool context. The regional species pool is defined as the set of regionally distributed species that could potentially colonize a given locality within that region, and it is a fundamental unit in biogeography and community ecology [63–66]. For instance, when species’ occurrences are sampled from a common species pool, the sign (+ vs. -) of an interspecific relationship can sometimes be inferred from co-occurrence data [67–70]. Species that rarely or never co-occur at the same sites may provide evidence of competitive exclusion, while frequent co-occurrence may provide evidence of positive interactions such as nest associations. But these co-occurrence patterns can easily be conflated with environmental filtering if the spatial scale of a study is large enough to effectively combine two or more regional pools. It is therefore critical to define the regional species pool in an objective and explicit manner. Network modules and multivariate clusters are both reasonable approximations of regional species pools [17] but as the *C* results show, they are not equivalent and will likely lead to different outcomes in studies of community assembly.

### Biotic interactions

Discrepancy in the *C* results between modules and PAM clusters begs an obvious question: which method is preferable? We suggest the nest associate results provide a meaningful context to address this question. Network modules were clearly superior to PAM clusters in preserving pairwise associations between the nest building Bluehead Chub and its nest associates. At all three spatial scales, the six documented nest associates were assigned to the same network module as the nest host (Table 2). However, when fishes from the same river basins were partitioned among an equivalent number of PAM clusters (*k* = no. modules) at the HU-2 and HU-4 scales, no more than one-half of the nest associates were placed in the same cluster as the host. The complete nest associate complex was only maintained in a single cluster at the HU-0 scale and this was likely due to spatial clustering within the large Mississippi River Basin, in which the Ohio River Basin and its fish fauna comprised a distinct biogeographic region. When the number of PAM clusters was not constrained to equal the number of modules (optimal *k* determined from the gap statistic) in the HU-4 scale Kanawha River Basin, the entire nest associate complex was preserved in a single cluster (Table 2). However, this was potentially an artifact of the smaller number of groups included in the optimal PAM solution; the odds of assigning the complete 7-species nest associate complex to a single group were logically greater when the Kanawha River fishes were partitioned among two PAM clusters, rather than four network modules (Table 1). Thus, we suggest that network modules may be superior to non-hierarchical, multivariate clustering tools, such as the PAM clusters used here, when the objective is to identify species linked by positive interactions.

Co-occurrence effect sizes further emphasize the importance of the nest associate results. At each of the three spatial scales, effect sizes within the nest associate complex were conspicuously larger than average effect sizes throughout the entire network (see Table 2 and Fig 4C). This result is intriguing because it shows that strong positive interactions among species can potentially be detected at very coarse scales. Even at the complete Mississippi River Basin (HU-0) scale, the mean effect size among nest associates was seven-fold larger than the mean effect size for the entire network. Our results now join a growing number of studies that have shown, on both theoretical [24] and empirical grounds [11,71,72], that co-occurrence data can be used to detect positive interactions across a range of spatial scales.

Moving forward, a significant challenge will be to determine which of the many remaining network links reflect biotic interactions and which reflect species that occur in the same habitats but are not interactive *per se*. For example, the complete HU-4 scale Kanawha River fish network includes 909 within-module links (summed among the four modules) and 704 among-module links (Fig 4A). Focusing exclusively on the module that includes the Bluehead Chub (green module in Fig 4A) reveals that the nest associate complex is central to the structure of the module; the force-directed layout used to build this network graph placed the most highly connected nodes near the center (Fig 4B). But the same graph also shows that the six nest associate links account for a tiny fraction of the 503 links within the module. Clearly, many other factors must have an influence on fish coexistence in the Kanawha River Basin.

We suggest that several non-exclusive strategies may be helpful in sorting through the large number of unexamined network links. First, effect sizes for all species pairs could be ranked and a significance threshold used to remove relatively weak links, prior to building and analyzing the networks [11]. This process would be straightforward because the probabilistic co-occurrence model used here to calculate effect sizes also includes a significance testing algorithm (see [59,60]). In a similar way, replacing the species’ presence-absence data used in each of our analyses (see Fig 1) with species’ abundance data (density estimates or relative abundances) might enhance our ability to detect and characterize species’ interactions. Presence-absence data, which are more readily available than abundance data, have been used extensively in traditional community ecology research (e.g., [73–75]) as well as more recent network-based studies (e.g., [16,72,76]). But turnover in species’ composition is rarely a binary or punctuated event. Rather, the addition or loss of a species from a local community is most often the result of a gradual increase or decrease in population size. Abundance data may therefore offer greater power to characterize interspecific relationships or to detect subtle changes in them [77–80].

Second, functional traits could be appended to species within the networks and used to search for traits-based patterns within or among modules [3,81]. For instance, the Bluehead Chub nest associate complex is particularly well-documented, but other stream fishes exhibit similar nest building and/or association behaviors [36,82]. It is therefore likely that analogous functional trait patterns or ‘motifs’ (*sensu* [83]) may occur within the network modules. In the Kanawha River Basin example, a comparable nest association motif might exist in the ‘purple’ module of Fig 4A, where another *Nocomis* nest builder, the River Chub (*Nocomis micropogon*), is prevalent. Recognition of repetitive functional motifs among modules might even be an effective way to guide the formulation and testing of hypotheses on other instances of positive interactions. The most likely interactions, as inferred from network links and species’ traits, could be identified, then tested through direct observational studies.

Finally, we note that the addition of phylogenetic information could resolve some of the remaining uncertainties regarding modular structure and species’ interactions. For example, if a recurring, convergent pattern of functional motifs is detected among network modules, a logical next step would be to test for a parallel pattern of phylogenetic overdispersion among species within the modules. This would test the hypothesis that contemporary functional motifs are attributable to historical partitioning (among modules) of closely related species that play similar ecological roles [84,85]. Alternatively, if species’ functional roles within modules are clustered, such that each module has a unique (rather than repeated) functional profile, then a similar pattern of phylogenetic conservatism could serve as a simple, historical null model of network structure [86].

In future work, we hope to pursue each of these ideas. But for the moment, we submit that careful consideration of spatial scale will be necessary to advance ecological network analysis. Our tests of spatial scaling provided modest support for the hypothesis that at larger scales, network modules are more likely to reflect biogeographical provinces than subgroups of interactive species. However, examination of the Bluehead Chub nest associates complex suggested that network modules may more useful than multivariate clusters for characterizing positive interspecific relationships. Furthermore, modules may be capable of detecting positive interactions across a wide range of spatial scales.
